# Syrian crises effect on specialty choice and the decision to work in the country among residents of six major hospitals in Syria, Damascus

**DOI:** 10.1371/journal.pone.0295310

**Published:** 2024-02-08

**Authors:** Jameel Soqia, Laila Yakoub-Agha, Lama Mohamad, Rawan Alhomsi, Mohamad Ashraf Shamaa, Albaraa Yazbek, Bayan Alsaid

**Affiliations:** Laboratory of Anatomy, Faculty of Medicine, Damascus University, Damascus, Syria; St John’s University, UNITED STATES

## Abstract

Assessing the impact of war on medical residents’ specialty choices and migration decisions is critical to ensure the sustainability of healthcare systems worldwide. This study aimed to evaluate the effect of the Syrian crisis on specialty choices, related factors, and decisions to work in Syria among residents of six major university hospitals in Damascus. A cross-sectional study was conducted using a validated questionnaire from 20/4/2022 to 20/5/2022, including all eligible residents with no missing data. The questionnaire was comprised of 68 items, and residents were divided into two groups: group 1 included residents who made their specialty choice after the end of the military war in Damascus 2018, while group 2 included residents who made their specialty choice (the point of submitting their lists and applying for residency) during the war. A total of 370 residents were included, with 38.4% females and 61.6% males. Our findings revealed that 30% of residents preferred working in Syria, while 43.5% preferred working abroad. The factor of a "safer and more stable life" was significantly higher in Group 1 than in Group 2 (3.86>3.5, p-value = 0.026). Additionally, Group 1 residents were more likely to choose radiology, pathology, laboratory, and psychiatry specialization as their specialties, while choosing surgical specializations and hematology decreased compared to Group 2 (p-value<0.05). Factors related to social life were rated higher by group 1 (mean = 3.31) than by group 2 (mean = 2.27, Standard deviation = 0.19, p-value = 0.002). Moreover, the factor of "a specialization to facilitate traveling abroad" was significantly higher in Group 1 (2.69>2.21, Standard deviation = 0.22, p-value = 0.033). The Syrian crisis and its economic aftermath have influenced residents’ specialty choices and practice locations. Even after the war’s end, the high level of migration intentions could negatively affect the quality of provided healthcare services.

## Introduction

Understanding how wars and crises influence the specialty choices of doctors and residents is vital for developing robust and sustainable healthcare systems that can address any potential shortages or deficiencies in particular fields [1]. As medical specialization and residency training continues to evolve, it is increasingly recognized as a critical topic for study and debate, particularly in terms of its impact on the planning and organizing of healthcare systems [2]. By examining the factors that affect these decisions, we can better anticipate and mitigate the effects of geopolitical instability on the provision of healthcare services.

The pervasive insecurity and violence that have characterized the Syrian conflict have undoubtedly contributed to the migration of healthcare workers and residents seeking safer and more stable living conditions, thus the Syrian crisis affected not only Syria’s health care systems but also its neighboring countries’ health care systems of Lebanon, Jordan, Iraq, Egypt and Turkey extending to European countries as well [3]. Our study highlights the ongoing challenges facing the healthcare system in Syria and emphasizes the need for sustained efforts to address these challenges and rebuild a functional healthcare system. There have been many factors that resulted in an uneven distribution of health workforce capacity across all specialties and areas where health care is needed, like damage to Syria’s healthcare system infrastructure, the overworked system, the lack of opportunities in the job market, financial concerns and the loss of many educators, doctors, and health care workers [4, 5]. In June 2018, Syrian state solidified its control over the areas of Damascus and its surroundings, and the military actions were stopped in all areas around the capital after six years of conflict [6]. In the following years, Syrians suffered from a socioeconomic crisis that resulted in a severe deterioration of living conditions which has also significantly eroded the health system [4].

Physicians’ migration is a common phenomenon that mainly occurs from low-income countries, which may lack the necessary equipment and resources for health care, to more developed and equipped countries [7, 8]. However, there are no research papers or data that have studied how the crisis or wartime affects the choice of medical specialty. This is an important topic to explore, as the need for physicians in wartime and crisis situations is undeniable [9, 10].

In a previous Syrian study that studied medical students’ willingness towards migration, 78.7% of participants were reportedly interested in working and migrating outside the country, and obtaining a visa was the most common obstacle [5].

During the economic crisis in Greece, studies showed that most of the medical students were planning to continue their medical specialty outside Greece, like Germany and the UK, which would cause a huge shortage in the workforce [10, 11]. Foreign training is the most important factor influencing migration intentions of medical graduates. This was studied, but less noted in times of conflict or countries during wars [8, 12].

The Syrian medical education for undergraduates ends within six years. Graduates who choose to specialize must list their specialty choices in the order of preference. Later, those lists are submitted to the residency institutions, in which they filter those choices according to students’ graduation scores, which finally leads the graduates to be allocated to their specialty. The duration of the specialization period in Syria can vary depending on the type of medical specialty, with programs ranging from 4 to 6 years in length. Resident who apply and are accepted into residency programs at university hospitals cannot change their specialty and must apply the same year they graduate.

This study was designed to evaluate and understand the Syrian crisis effect on residents’ choice of specialization and to measure what factors influenced this choice among residents of Syria.

## Methods

### Study design

This study was a cross-sectional exploration into the impact of the Syrian crisis on medical specialty choices, and related factors of resident doctors in Damascus university hospitals. It evaluated Damascus university residents’ decision to work in the country or to migrate and the effect of the Syrian crisis on it. Additionally, this study followed STROBE guideline for cross-sectional studies.

### Participants and setting

The questionnaire was distributed to residents to residents of all years of different specialties in the following hospitals: Al Assad University Hospital, Al Mouwasat University Hospital, Children University Hospital, University Hospital of Dermatology and Venereology in Damascus, University Hospital of Obstetrics and Gynecology in Damascus, Al Bairouni University Hospital. Residents were divided into 2 groups. Group 1 included residents in the first, second, and third year of their residency, as these residents made their medical specialty choice after the end of the military war that ended in 2018 (2021, 2020, and 2019, respectively). Group 2 included residents in the fourth, fifth, and sixth year of their residency, as these residents made their medical specialty choice before the end of the military war that ended in 2018 (2018, 2017, and 2016, respectively). Residents of university hospitals cannot change their specialty after applying and being accepted into residency programs, and they must apply at the same year of their graduation, according to the Syrian Minister of Higher Education and Scientific Research and Damascus University internal system.

### Sample

We chose residents to make this study because they had already made their choice, unlike medical students who may change their choice after the study. In Damascus, there are six university hospitals that are widely recognized as the largest medical facilities in Syria. However, it’s worth noting that the residents of these hospitals are affiliated with Damascus University as a whole, rather than any specific hospital. Therefore, they often rotate among the hospitals based on their training program. To ensure that our sample is representative, we had computed it based on the university departments and their total number of residents, such as the General Surgery department.

Based on the 2022 census data from the Syrian Minister of Higher Education and Scientific Research and Damascus University, the estimated number of residents across all Damascus University hospitals was 1595. The sample size was calculated using a Raosoft, an online sample size calculator. Confidence interval (CI) was set at 95%, margin of error at 5% and with population size of 1595. A sample size of at least 311 was needed.

Furthermore, to ensure that our study encompasses all specializations, we had determined the necessary number of participants from each specialization based on the overall sample size calculation. You can refer to Table 1 for the total number of residents in Damascus University, as well as the minimum required number and the sample size for our study.

**Table 1:** Participants’ specializations distribution and sample calculation.

Specialization	Total number of residents	Minimum required number	Sample size for our study
Pediatrics	120	24	28
Hematology	20	4	5
Obstetrics and Gynecology	42	7	8
Pediatric Surgery	7	2	3
Thoracic Surgery	24	5	5
Plastic Surgery	26	5	7
Orthopedics	49	10	12
Radiology	243	46	46
Clinical Pathology	56	10	12
Pulmonology	29	6	6
Gastroenterology	29	6	9
Anesthesiology	87	15	15
Dermatology	58	11	14
Cardiac Surgery	42	9	12
Nephrology	25	5	5
General Internal Medicine	235	45	51
Cardiology	26	5	6
Neurology	25	5	7
Oncology	36	7	10
Rheumatology	23	5	8
Endocrinology	18	4	4
Laboratory	53	11	11
Emergency Medicine	10	2	3
Psychiatry	45	9	12
Infectious Diseases Medicine	19	4	6
General Surgery Medicine	68	13	18
Urinary Surgery	33	7	8
Neurosurgery	25	5	7
Vascular Surgery	29	6	8
Ophthalmology	41	8	11
Otolaryngology	46	9	12
Radiotherapy	5	1	1
**Total**	**1595**	**311**	**370**

A total of 370 resident doctors of 32 specialties (Table 1) from all years available were registered in the study; residents were asked to answer the questionnaire voluntarily, and were approached using simple random sampling (SRS) technique. Also, each of the 32 specialties were represented correctly as the sample calculation required (Table 1).

### Questionnaire

Full questionnaires were mentioned in S1 File (English), and S2 File (Arabic).

The questionnaire was designed to assess the identified factors influencing the selection of a medical specialty. The process by which the instruments were developed was as follows: a literature review about instruments related to the construct of interest was performed in the major databases; a group of medical students and an experienced doctor assessed and picked out the specified questionnaires; appropriate elements were chosen from a validated questionnaire in a previous study [13]. It was first prepared in English, translated into Arabic (the native language) by language experts, and re-translated into English for consistency in the meaning of words and concepts. A pilot study was carried out with 50 residents, and the questionnaire was checked according to the primary statistical study. Also, the survey was tested for reliability using Cronbach’s Alpha test. Internal stability of (0.789).

The final version included 68 items divided into two sections: a) Demographic features (20 items), including specialty, specialty year, age, gender, college, family situation, information about family education, whether or not they took part in college activities and in scientific activities outside the college, when did they choose their specialty, and ultimately, how were they affected by their doctors and teacher. The second section (b) consisted of factors involved in specialty selection (23 items) and used a Likert scale, from non-determinant (1) to most determinant (5), and whether they are satisfied with their specialty and if it was their first choice. In addition to the factors related to not getting into the specialty, they were initially looking for. This contains 12 factors using the same scale as the one used for the previous part, then there are 10 factors associated with where they decided to work once, they completed their residency period, using the same scale. Finally, the response time of the instrument was approximately 10 minutes.

### Data analysis

The data entry and analysis were performed using Statistical Package for Social Sciences software package (SPSS Inc., Chicago, IL, USA) version 23. Descriptive statistics were obtained. For categorical variables, chi-square independence tests, and T-test analyses were performed with 95% confidence intervals. A P value of <0.05 was considered statistically significant.

### Ethical aspects

The study was in compliance with the Declaration of Helsinki for research involving human subjects. The Ethical Committee approved this study in the Faculty of Medicine at Damascus University, Syria (1517, 2/3/2022). Before participants filled out the questionnaire for the study, both verbal and written informed consent were obtained and documented. To ensure consent was obtained, we added a question specifically asking for it before starting the questionnaire. Furthermore, the ethical committee at Damascus University approved this consent procedure.

## Results

The study included a total of 370 residents. The gender distribution was a split 40:60 in the general resident population, namely 142 females (38.4%) and 228 males (61.6%). Other statistics are shown in Table 2.

**Table 2:** Sample characteristics.

Variables		Frequency		Percent	
		Group 1 (residents of the first three years)	Group 2 (residents of the last three years)	Group 1 (residents of the first three years)	Group 2 (residents of the last three years)
Total number		324	46	87.6	12.4
Gender distribution	Females	127	15	39.1	32.7
	Males	197	31	60.9	67.3
Year of specialization	First year	101		31.1	
	Second year	141		43.5	
	Third year	82		25.3	
	Fourth year		28		60.8
	Fifth year		13		28.2
	Sixth year		5		10.8
Age	Less than 25 years	50	0	15.4	0
	Between 25–30 years	272	40	83.9	86.9
	Older than 30 years	2	6	0.06	13.0
Familial (Marital) Status	Single	287	32	88.5	69.5
	Married	37	14	11.4	30.4
	Divorced with children	0	0	0	0
	Divorced without children	0	0	0	0
Graduation college	Damascus University	222	27	68.5	58.6
	Al-Baath University	36	9	11.1	19.5
	University of Hama	14	1	4.3	2.1
	Tishreen University	16	4	4.9	8.6
	Tartous University	14	0	4.3	0
	Aleppo University	9	4	2.7	8.6
	Al-Fourat University	1	0	0.3	0
	Al-Andalus University	3	0	0.9	0
	SPU	3	0	0.9	0
	Al- Sham Private University	1	0	0.3	0
	Al-Kalamoon University	2	1	0.6	2.1
	others	3	0	0.9	0
Stage of deciding on specialization	Before college	23	2	7.0	4.3
	First three years of college	17	2	5.2	4.3
	Clinical years of college (last three years)	100	16	30.8	34.7
	After graduation	184	26	56.7	56.5
The influence of professors on the students’ decision of specialization	Positive influence	102	9	31.4	19.5
	Negative influence	12	2	3.7	4.3
	No influence	210	35	64.8	76

The number of residents who want to continue working in Syria was 111 (30%) with no desire to leave Syria, while the number of residents who want to continue working abroad was 161 (43.5%) with no desire to stay in Syria. Other residents had multiple desires.

There was no significant difference between the residents’ desire to stay in Syria of the residents who reported being positively influenced by a teacher, those who reported being negatively influenced, and those who were not influenced at all, X^2^ (2, N = 370) = 1.94, p = 0.379. Moreover, the influence of professors on the residents’ specializations was not significantly different between residents in group 1 and group 2, X^2^ (62, N = 370) = 66.2, p = 0.3.

Factors that most of the residents agreed to have an effect on their desired after-graduation workplace: the preference of the type of life and the quality of the practical life. Other factors’ means are shown in Table 3.

**Table 3:** Factors affecting on residents’ decision in choosing the work place after specialization and its association with group 1 and 2.

Factors	N	Mean	Std	Mean of group1 and 2		Std for group 1 and 2		t	sig	std
Ability to maintain social relationships	370	3.295	1.1744	3.333	3.022	1.1565	1.2735	1.635	0.099	0.18
The sense of belonging	370	3.176	1.1424	3.213	2.913	1.1298	1.2079	1.651	0.1	0.17
Lack of specialists	370	2.884	1.2142	2.843	3.174	1.2024	1.2702	1.752	0.08	0.19
Greater opportunities for my kids	370	3.011	1.2141	3.046	2.761	1.2143	1.1960	1.512	0.13	0.19
Greater Opportunities for my partner	370	3.073	1.1465	3.105	2.848	1.1409	1.1732	1.466	0.14	0.17
An opportunity to gain money	370	3.630	1.0773	3.617	3.717	1.0740	1.1088	0.589	0.55	0.16
To continue my education	370	3.624	1.0830	3.651	3.435	1.0726	1.1480	1.310	0.19	0.17
For safer and more stable life	370	3.795	1.0469	3.836	3.500	1.0235	1.1690	2.074	0.039	0.16
The preference of the type of life	370	3.822	1.1240	3.849	3.630	1.1039	1.2536	1.23	0.21	0.17
The quality of the practical life	370	3.824	1.0815	3.833	3.696	1.0659	1.1901	0.82	0.41	0.17

Mean from 1 to 1.8 represents (strongly disagree), from 1.81 to 2.6 represents (disagree), from 2.61 to 3.4 represents (true to some extent), from 3.41 to 4.20 represents (agree) and from 4.21 to 5 represents (strongly agree).

These factors’ influence on the residents was tested among two groups of residents; group 1 who made their specialty decision after the end of the actual war (residents in their first 3 years of residency) and group 2 who made their specialty decision before the end of the actual war (residents in their last three years of residency). The only factor which was significantly different between group 1 and 2 as shown in Table 3 is “For safer and more stable life”, t (363) = 2.074, P = 0.039, and std = 0.1621; group 1 residents’ agreement to this factor as an influencing factor was higher than group2 (3.86>3.5).

Residents’ desire to continue working in Syria showed no significant difference between group 1 and group 2, X^2^ (1, N = 370) = 0.009, p = 0.52; around 56% of both groups reported that staying in Syria is a possible option for them. Similarly, there was no significant difference between group 1 residents’ desire to continue their career outside Syria and group 2, X^2^ (1, N = 370) = 0.115 p = 0.44; around 70% of both groups reported that leaving the country is a possible choice for them.

There was a significant difference between the chosen specialty of the group 1 residents and the group 2, X^2^ (31, N = 370) = 66.7, p<0.05; There was a significant decrease in the number of residents specializing in hematology, pulmonology, rheumatology, ophthalmology, and plastic surgery in group 1 compared to group 2, while there was a significant increase in the number of residents specializing radiology in group 1 compared to group 2. No residents in group 2 specialized in the following specialization (orthopedics, cardiology, oncology, endocrinology, laboratory, emergency medicine, and psychiatry) in contrast to group 1. Moreover, when comparing the number of residents specializing in surgery specializations (in general), there was a significant decrease in the number of surgeons in group 1 compared to group 2 (as shown in Table 4). However, the chosen specialty of the residents did not have a significant relation with neither their desire to continue working in Syria (X^2^ (31, N = 370) = 32.7, p = 0.38) nor their desire to continue working abroad (X^2^ (31, N = 389) = 30.7, p = 0.48).

**Table 4:** A comparison between group 1 and 2 specializations.

Specialization	Frequency	
	Group 1 (residents of the first three years)	Group 2 (residents of the last three years)
Pediatrics	27_a_	1_a_
Hematology	2_a_	3_b_
Obstetrics and Gynecology	6_a_	2_a_
Pediatric Surgery	2_a_	1_a_
Thoracic Surgery	4_a_	1_a_
Plastic Surgery	3_a_	4_b_
Orthopedics	12_a_	0^1^
Radiology	45_a_	1_b_
Clinical Pathology	11_a_	1_a_
Pulmonology	3_a_	3_b_
Gastroenterology	7_a_	2_a_
Anesthesiology	14_a_	1_a_
Dermatology	12_a_	2_a_
Cardiac Surgery	9_a_	3_a_
Nephrology	4_a_	1_a_
General Internal Medicine	48_a_	3_a_
Cardiology	6_a_	0^1^
Neurology	6_a_	1_a_
Oncology	10_a_	0^1^
Rheumatology	5_a_	3_b_
Endocrinology	4_a_	0^1^
Laboratory	11_a_	0^1^
Emergency Medicine	3_a_	0^1^
Psychiatric	12_a_	0^1^
Infectious Diseases Medicine	5_a_	1_a_
General Surgery Medicine	15_a_	3_a_
Urinary Surgery	6_a_	2_a_
Neurosurgery	^5a^	^2a^
Vascular Surgery	5_a_	_3b_
Ophthalmology	_10a_	_1a_
Otolaryngology	1_1a_	1_a_
Radiotherapy	_1a_	0^1^
Surgery specializations	61_a_	19_b_
**Total**	324	46

Note: values in the same raw or subtable not sharing the same subscript are significantly different at p<0.05 in the two-sided test of equality for column proportions. Cells with no subscript are not included in the test. Test assume equal variances. 1. This category is not used in comparisons because its column proportion is equal to zero or one. 2. Tests are adjusted for all pairwise comparisons within a row of each innermost subtable using the Bonferroni correction

Independent T-test showed a significant difference in the agreement of group 1 and group 2 to some factors that had an influence on the residents’ desirable specialization. Choosing “a specialization that facilitates traveling abroad” was significantly different between the two groups, t (368) = 2.134, (P = 0.033, std = 0.2235); group1 had a higher mean than group 2 (2.69 and 2.21 in order). Additionally, all factors related to the social life (“a manageable specialization that guarantees a free time for the specialist”, “a specialization that enables the specialist to balance between work and family”, and “a specialization that enables the specialist to maintain a social life”) were significantly different between group1 and group 2, t (368) = 3.05, (p = 0.002, std = 0.19),]; group1 had a higher mean than group 2 (3.31>2.27). Moreover, the agreement to the factor “the length of the residency” was significantly higher in group 1 compared to group 2, and the agreement to the factor “the ability to deal with only one gender” was significantly higher in the group 2 compared to the group 1. Other factors were not significantly different between groups, and the means are as shown in Table 5.

**Table 5:** Means of the influencing factors on the residents’ desirable specialty (group 1 and 2) and their association with group 1 and group2.

The affecting factors	group	N	means	std	t	sig	std
Personal interests	1	324	3.762	1.1440	-.373	.709	.1792
	2	46	3.826	1.0812			
Family pressure	1	324	2.086	1.2032	-1.941	.053	.1907
	2	46	2.457	1.2597			
A manageable specialization that guarantees a free time for the specialist	1	324	3.231	1.3625	2.116	.035	.2180
	2	46	2.761	1.4935			
A specialization that enables the specialist to balance between work and family	1	324	3.364	1.2942	2.994	.003	.2067
	2	46	2.739	1.3894			
A specialization that enables the specialist to maintain a social life	1	324	3.367	1.3206	3.275	.001	.2099
	2	46	2.674	1.3993			
Friends’ advice and guide	1	324	2.806	1.2301	.328	.743	.1934
	2	46	2.739	1.2371			
Professors’ advice and guidance	1	324	2.645	1.2836	.679	.497	.1999
	2	46	2.500	1.2065			
Lack of specialists	1	324	2.444	1.2978	-.029	.977	.2028
	2	46	2.457	1.2240			
The financial outcome	1	324	3.046	1.3100	.634	.527	.2053
	2	46	2.913	1.2619			
The reputation of the specialization	1	324	3.401	1.2662	1.058	.291	.2031
	2	46	3.174	1.4190			
Social standing	1	324	3.148	1.2552	-.814	.416	.1957
	2	46	3.304	1.1713			
Greater job opportunities	1	324	3.383	1.1915	.984	.326	.1901
	2	46	3.196	1.3101			
The length of the residency	1	324	2.997	1.2454	2.897	.004	.1941
	2	46	2.435	1.1285			
The ability to study subspecialty	1	324	3.386	1.2722	-.136	.892	.2005
	2	46	3.413	1.2750			
Greater research opportunities	1	324	3.000	1.2664	.330	.742	.1976
	2	46	2.935	1.1624			
The age of patients	1	324	2.704	1.2108	2.354	.019	.1894
	2	46	2.261	1.1242			
Scientifically rich specialty	1	324	3.602	1.2337	1.624	.105	.1966
	2	46	3.283	1.3443			
Common specialty diseases	1	324	3.608	1.1581	2.209	.028	.1852
	2	46	3.196	1.3101			
Satisfying Treatment results	1	324	3.386	1.2276	1.206	.229	.1938
	2	46	3.152	1.2466			
The experience of the professors	1	324	3.503	1.2529	.839	.402	.2000
	2	46	3.326	1.3342			
The effect of the social medias	1	324	2.546	1.1353	1.338	.182	.1785
	2	46	2.304	1.1327			
The ability to deal with only one gender	1	324	1.898	1.0284	-2.062	.040	.1669
	2	46	2.239	1.2505			
To facilitate traveling abroad	1	324	2.694	1.4324	2.134	.033	.2235
	2	46	2.217	1.3151			

Mean from 1 to 1.8 represents (strongly disagree), from 1.81 to 2.6 represents (disagree), from 2.61 to 3.4 represents (true to some extent), from 3.41 to 4.20 represents (agree) and from 4.21 to 5 represents (strongly agree).

As group 1 refers to residents in the first three years, and group 2 refers to residents in the last three years of medical school.

## Discussion

The Syrian crisis’s effect on both residents’ choice of specialty and whether to travel abroad was investigated in our study. Results collected from our residents’ sample demonstrated that the crisis affected their priorities and therefore specialty choosing process.

Conflict zones are characterized by a high level of violence, destruction, and insecurity, which can have a significant impact on the health and wellbeing of individuals living and working in these areas [4]. In particular, doctors and other healthcare professionals are often on the front lines of conflict, providing emergency medical care to those who have been injured or traumatized by the violence [4]. However, despite the importance of understanding the experiences of medical professionals in conflict zones, there is a lack of research on this topic, particularly with regard to the Syrian crisis [4]. While measuring the exposure of conflict for research participants could provide valuable insights into the impact of war on individual health outcomes, it may not always be feasible or ethical to do so in conflict zones where the safety of participants cannot be guaranteed. Thus, it is important for researchers to find alternative ways of exploring the experiences of doctors and other healthcare professionals in conflict zones, in order to better understand the dynamics of the conflict and its impact on those working in the healthcare sector.

Starting with group 2, who chose their specialty during the time of military actions in Syria, our results were different compared to another local study done in 2016. Bisher et al. stated that the top three factors affecting participants’ choice were flexibility of work schedule, ease of finding jobs after specializing, and training hours [5]. Meanwhile, our study cited personal interests, the ability to subspecialize, and the professors’ experience. Shifting to group 1, who made their decision after the end of the war and during the economic crisis. The fundamental criteria for selecting a specific medical specialization were personal interests, a scientifically rich specialty with common diseases, professors’ experience, and specialization with a controllable lifestyle. These findings were supported by the results of a study done in Greece during a similar economic crisis [10]. Another study done in Spain agreed with our results relating great importance to a controllable lifestyle, working hours, recognition by patients, and prestige among colleagues [14].

Residents who selected their specialty after the war (group 1) ranked social life and program duration with greater importance compared to residents who selected their specialty during the war (group 2). This might explain why group 1 showed a higher percentage specializing in radiology, pathology, laboratory, and psychiatry specialization, whereas group 2 was more likely to give precedence to surgery and hematology. This finding is consistent with a recent study conducted in Japan illustrating that there is an increase in young physicians who place greater emphasis on lifestyle while choosing their specialties [15]. Prioritizing social life could help prevent burnout, which was shown to be high among Syrian residents [16]. It is worth noticing that the choice of specialty was not affected by the desired area of practice, i.e., in Syria or abroad.

Residents who selected their specialty during the war (group 2) showed a greater emphasis on choosing a specialty that facilitates traveling compared to Residents who selected their specialty after the war (group 1). This could indicate that the main driving factors for residents to migrate are the economic crisis, lack of resources, and the enormous destruction in the Syrian healthcare system as stated in a recent meta-analysis [16].

Around 70% of both groups, unfortunately, considered continuing their career outside Syria. The highly prevalent intention to migrate is similar to a study conducted in Syria in 2016, which found 78% of their participants willing to migrate for specialization [5]. Other studies discussing migration intention in times of crisis showed overall agreement with our results. In Iraq, 49.5% of doctors intend to migrate [17] whereas 95.5% of medical students in Lebanon plan to travel abroad either for specialty or subspecialty training [9]. Additionally, 55% of junior doctors in Portugal [18], 85% from Greece [10], 84% from Serbia [19], and 72% from Gaza [20] considered working abroad.

There are several possible explanations for the wide range of percentages in the rate of migration-intention among the previously mentioned countries. Firstly, the differences in the target population, e.g., we surveyed residents and not medical students. Secondly, there are huge differences between the countries mentioned above in terms of geography, safety level, and socioeconomic status. Another priority was the preference to deal with one gender, which was cited more by group 1. On this basis, future research could discuss the crisis’s effect on gender norms among Syrian medical students and residents, nevertheless, factors affecting both groups of residents’ choice of career place were the quality of the practical life and education, a controllable lifestyle, and an opportunity to earn money. This finding is in line with Santric-Milicevic et al. who stated that the main factors that are pushing medical students to consider working abroad were satisfactory employment opportunities and the quality of life [21]. Same results were also found in Pakistan [22], and Ireland [23]. Our findings showed higher importance tied to a more stable and safer life among group 1, which needs further exploration through future studies.

More than half of our participants (60%) cited the absence of teachers’ influence on their specialty choice. This was similar among both resident groups (1 and 2). Whether influenced by a teacher or not, residents’ migration intention was not affected. This observation is different from a previous study done in France where the impact of both teaching and teachers on medical students’ career choices was significant [24]. Furthermore, another study in Canada illustrated that exposure to role models in a particular clinical field was strongly correlated with medical students’ choice of a clinical field for residency training [25].

The low influence in our study is possibly due to the diminished possibility of interpersonal relationships with professors caused by the large students’ numbers and the shortage of academic staff members.

### Policy implications

The policy implications of our study findings are twofold. First, they suggest that there is an urgent need for interventions to support and retain medical professionals in Syria, especially those who are interested in surgical and hematological specialties that are critical for providing trauma care and blood transfusion services in conflict zones. Such interventions could include improving working conditions and security measures for doctors and residents, providing incentives and recognition for their work, enhancing training opportunities and mentorship programs, and facilitating access to resources and equipment. Second, they indicate that there is a potential for collaboration and exchange between Syrian medical professionals and those in other countries who share similar interests and challenges. Such collaboration could foster mutual learning and support among doctors and residents across different contexts and cultures, as well as contribute to global health research and practice. Therefore, we recommend that policymakers and stakeholders at national and international levels should consider developing policies and programs that address these issues and promote the retention and development of medical professionals in Syria. Our study findings have two policy implications. First, they imply that interventions to support and retain medical professionals in Syria are urgently needed, especially for those who specialize in surgery and hematology, which are essential for trauma care and blood transfusion services in conflict zones. Possible interventions include improving working conditions and security measures, providing incentives and recognition, enhancing training and mentorship, and facilitating access to resources and equipment. Second, they suggest that there is an opportunity for collaboration and exchange between Syrian medical professionals and their counterparts in other countries who face similar interests and challenges. Such collaboration could enhance mutual learning and support among doctors and residents from different contexts and cultures, as well as advance global health research and practice. Therefore, we advise that policymakers and stakeholders at national and international levels should develop policies and programs that address these issues and foster the retention and development of medical professionals in Syria.

### Limitations

Several limitations are noticed in this study. First, the number of group 2 participants was smaller than group 1, which could impair generalizability. Second, although our study population represents residents from a wide range of medical schools in Syria, only residents training in Damascus hospitals who are affiliated with the Ministry of Higher Education (MHE) residency programs were included. Residents of other governorates and/or different programs differ in terms of socioeconomic status and perceptions of medical specialty, thus career plans due to the variation in war consequences between different areas of the country. Another limitation of studying the impact of conflict on healthcare professionals is the difficulty of measuring exposure to violence and trauma in a safe and ethical manner. This can make it challenging to fully understand the specific experiences and needs of medical professionals working in conflict zones. This implies the need for larger-scale studies with a more diverse set of institutes to minimize bias and improve generalizability, which was limited by the convenient sampling method. Also, we acknowledge that a limitation of our study is the lack of official data available on the gender distribution of residents in Syria, which prevents us from making a direct comparison between the gender distribution of our sample population and the general resident population. One limitation of this study is that the two groups of residents who graduated before and after the war may not be completely comparable in terms of their specialty preferences. The post-war group may still have concerns about the status of Syria, which could affect their decision to stay or migrate. To fully answer the research question, it would be ideal to have a longer time gap between the two groups. However, given the urgency and relevance of this study to address the current immigration problem of Syrian doctors and shortage of specific specializations, we decided to use the available data and adjust for potential confounders in our analysis. Future studies should explore this question with a larger sample size and a longer follow-up period.

## Conclusion

As expected by the research team, the Syrian crisis throughout the war and the subsequent economic sequelae influenced our study participants’ specialty choice and location of the practice. There was a significant difference in the perceived priorities between residents who chose their specialty before the war and residents who made their specialty choice after the war. Even after the end of the Syrian war, during the economic crisis, migration intention remained high. This alarming level of resident migration out of Syria will likely to disrupt the healthcare system and diminish the quality of services provided.

## Supporting information

S1 File This questionnaire aims to collect data about factors affecting the choice of medical specialty. (PDF)

S2 File (PDF)
