# Supplementary material for: Syrian crises effect on specialty choice and the decision to work in the country among residents of six major hospitals in Syria, Damascus
Source: PLoS One. 2024 Feb 8;19(2):e0295310. doi: 10.1371/journal.pone.0295310 (PMC10852239; doi:10.1371/journal.pone.0295310)
Supplement: S1 File — (PDF) [file pone.0295310.s001.pdf]

This questionnaire aims to collect data about factors affecting the choice of medical specialty.

Demographic information

• **Specialty**:	• **Specialty year**:	• **Age:**
• **Gender:**<br>○ Female.<br>○ Male.	• **University**:	• **place of residence before specialty:**
• **Marital status:**<br>○Single.<br>○Married.<br>○ divorced with children.<br>○ divorced with no children.	• **If you are married, does your husband work in the medical field?**<br>○ Yes.<br>○ No.	• **Education level of mother:**<br>○ Less than secondary education.<br>○ Secondary education.<br>○ University degree.<br>○ Postgraduate.
• **Are your parents doctors?**<br>○ No.<br>○ Yes, both of my parents.<br>○ Yes, only my father.<br>○ Yes, only my mother.	• **Medical specialty of your mother:**	• **Medical specialty of your father:**

<table>
<tr><td>• Have you participated in a scientific research during your medical studies؟
○ Yes.
○ No.</td><td colspan="2">• Have you participated in teaching activities during your medical studies؟
○ Yes, in and outside the university.
○ Yes, only inside the university.
○ Yes, only outside the university.
○ No.</td></tr>
<tr><td>• Have you participated in study groups or congress's related to the specialty you are interested؟
○ Yes.
○ No.</td><td colspan="2">• Did you participate in a volunteer team during your university studies؟
○ Yes, a team to write lectures in my specialty itself.
○ Yes, a team to write lectures in another specialty.
○ Yes, a team in another field.
○ No.</td></tr>
<tr><td>• At what stage did you decide your specialty?
○ Prior to entering Medical School.
○ Before the clinical internship (first 3 years).
○ During the clinical internship (4/5/6).
○ After the clinical internship.</td><td>• When choosing a specialty:
○ My opinion changed a lot.
○ My opinion remained unchanged.
○ I didn't choose, I specialized according to the university average.</td><td>• In terms of professors' influence on your choice:
○ I was positively influenced by a professor at my university, which prompted me to choose a major.
○ I was negatively influenced by a professor at my university, so I changed my mind.
○ I wasn't influenced.</td></tr>
</table>

Evaluate the factors affecting your choice of specialty:

Factors	Strongly agree	Agree	neutral	disagree	Strongly disagree
Personal interest.					
Family pressure.					
Controllable, I guarantee having free time.					
Ability to balance between work and family.					
Have a social life.					
Advised and guided by a friend.					
Advised by doctors and professors.					
Lack of specialties.					
Financial income.					
The reputation of specialty.					
Social status (importance).					
Greater job opportunities					
Period of residency					
Ability to study subspecialty.					
Greater research opportunities.					
Age category of patients					
Scientifically rich specialty.					
Common specialty diseases.					
Satisfying treatment results.					
The experience of the professors					
The influence of social medial					
The ability to deal with only one gender.					
The specialty facilitates travel procedures.					

- **Are you satisfied with your specialty ?**
  - **Yes.**
  - **No.**

- **What specialty was your first desire?**

- **Is your specialty your first desire?**
  - **Yes.**
  - **No.**

- **If the answer is no, identify the factors that prevented you from obtaining your first desire:**

Factors	Strongly agree	agree	neutral	disagree	Strongly disagree
Graduation academic score.					
I believe in the important role of the doctor's gender.					
The society believe in the important role of the doctor's gender.					
The specialty does not provide time for me and my family.					
Poor financial income					
Specialty Monopoly .					
high work pressure					
The cost of the clinic					
Patient's gender					
Poor patient's satisfaction					
Routine work					
Partner refusal					

- **Where will you work after completing the specialization years؟**
  - Countryside.
  - City.
  - Arabian country.
  - Foreign country.

- **What are the factors affecting the choice of working place after specialization?**

Factors	Strongly agree	agree	neutral	disagree	Strongly disagree
Keeping the social relationships					
The sense of belonging					
Lack of specialists					
Better opportunities for children to learn.					
Partner opportunities (work/ study)					
Greater opportunities to gain money					
To continue my education					
More stability and security life (safety)					
Lifestyle Preference					
The quality of practical life.					
