# Supplementary material for: Syrian crises effect on specialty choice and the decision to work in the country among residents of six major hospitals in Syria, Damascus
Source: PLoS One. 2024 Feb 8;19(2):e0295310. doi: 10.1371/journal.pone.0295310 (PMC10852239; doi:10.1371/journal.pone.0295310)
Supplement: S2 File — (PDF) [file pone.0295310.s002.pdf]

جامعة دمشق

كلية الطب البشري

إن الاستمارة التالية تهدف إلى جمع بيانات حول العوامل المؤثرة في اختيار الطلاب لاختصاصهم الطبي

اختصاصي الطبي : .... سنة الاختصاص : ....

معلومات عامة

- العمر: ....

- الجنس:
  - ○ أنثى
  - ○ ذكر

- درست في جامعة: ....

مكان الإقامة ما قبل الجامعة: ....

- الحالة العائلية:
  - ○ أعزب/عزباء
  - ○ متزوج/ة
  - ○ منفصل/ة "لدي أطفال"
  - ○ منفصل/ة "لا أطفال"

- في حال كنت متزوّج، هل زوجك يعمل في المجال الطبي؟
  - ○ نعم
  - ○ لا

- التخصص الطبي للأب: ....

- مستوى تعليم الأب:
  - ○ أقل من الشهادة الثانوية.
  - ○ الشهادة الثانوية.
  - ○ الشهادة الجامعية.
  - ○ الدراسات العليا.

- مستوى تعليم الأم:
  - ○أقل من الشهادة الثانوية.
  - ○الشهادة الثانوية.
  - ○الشهادة الجامعية.
  - ○الدراسات العليا.

- هل الوالدان طبيبان؟
  - ○ لا
  - ○ نعم كلاهما
  - ○ نعم، والدي فقط
  - ○ نعم، والدتي فقط

- التخصص الطبي للأم: ....

المرحلة الجامعية

- هل شاركت في أبحاث علميّة خلال دراستك؟
  - ○ نعم
  - ○ لا

- هل شاركت في مجموعات دراسيّة أو ساعدت في مؤتمرات متعلقة بالتخصص الذي تهتم به؟
  - ○ نعم
  - ○ لا

- هل شاركت في عملية التدريس خلال دراستك (ضمن الجامعة أي ضمن الحرم الجامعي، خارج الجامعة تتضمن أيضاً مواقع التواصل)؟
  - ○ نعم، في الجامعة وخارجها.
  - ○ نعم، في الجامعة فقط.
  - ○ نعم، خارج الجامعة فقط.
  - ○ لا.

- هل شاركت في أحد الفرق التطوعية خلال دراستك؟
  - ○ نعم، في فريق لكتابة المحاضرات، في نفس تخصصي الحالي.
  - ○ نعم، في فريق لكتابة المحاضرات، في تخصص آخر.
  - ○ نعم، في فريق تطوعي ضمن مجالات أخرى.
  - ○ لا.

- في أي مرحلة قررت اختيار تخصصك الحالي:
  - ○ قبل دخولي لكلية الطب.
  - ○ في مرحلة ما قبل التدريب السريري(أول 3 سنوات).
  - ○ في مرحلة التدريب السريري(6/5/4).
  - ○ في مرحلة ما بعد التدريب السريري (المفاضلة).

- خلال اختيارك للاختصاص:
  - ○ تغيّر رأيي كثيراً حتى استقر.
  - ○ ظلّ رأيي ثابتاً.
  - ○ لم أختر، تخصصت حسب معدلي الجامعي.

- بالنسبة لتأثير الأساتذة على اختيارك للاختصاص:
  - ○ تأثرت إيجاباً بأستاذٍ في جامعتي، مما دفعني لاختيار الاختصاص.
  - ○ تأثرت سلباً بأستاذ في جامعتي، مما جعلني أغير من رأي.
  - ○ لم أتأثر.

- قيم العوامل المؤثرة في قرارك: (١ غير موافق بشدة/ ٢ غير موافق/ ٣ حيادي/ ٤ موافق/ ٥ موافق بشدة)

العوامل	١	٢	٣	٤	٥
اهتمامات شخصية					
ضغط العائلة					
يمكن التحكم به فأضمن الوقت الخاص بي					
إمكانية الموازنة بين العمل والعائلة					
المحافظة على الحياة الاجتماعية					
توجيه ونصح من الأصدقاء					
توجيه ونصح من أطباء وأساتذة					
نقص المتخصصين					
المردود المادي					
سمعة الاختصاص والرغبة فيه					
المكانة الاجتماعية					
فرصة عمل أكبر					
مدة الإقامة					
إمكانية دراسة تحت اختصاص					
فرصة أكبر للبحث العلمي					
الفئة العمرية للمرضى					
اختصاص ثري علمياً					
الأمراض شائعة					
نتائج العلاج مرضية					
الأساتذة على درجة عالية من الخبرة					
تأثير مواقع التواصل					
إمكانية التعامل مع جنس واحد من المرضى					
اختصاصي لتسهيل إجراءات السفر					

- هل أنت راضٍ عن اختصاصك؟
  - نعم
  - لا

- هل اختصاصك الحالي يمثل رغبتك الأولى؟
  - نعم
  - لا

- في حال الإجابة ب لا، حدد العوامل التي منعتك من دخول الاختصاص المتمثل بالرغبة الأولى:

العوامل	١	٢	٣	٤	٥
المعدل الجامعي					
لاعتقادي بالدور الهام لجنس الطبيب					
لاعتقاد المجتمع بالدور الهام لجنس الطبيب					
لا يوفر الوقت لي ولعائلتي					
المردود المادي سيء					
احتكار الاختصاص					
الضغط العالي					
كلفة العيادة					
جنس المرضى					
رضى المرضى أقل لأن معظم أمراضه مزمنة					
طبيعة العمل الروتينية					
تجنباً لبعض الإجراءات المخالفة للدين					
رفض الزوج					

- أين ستعمل بعد إنهاء سنوات الاختصاص؟
  - الريف
  - المدينة
  - خارج البلاد(بلد عربي)
  - خارج البلاد(بلد أجنبي)

- وفقاً لاختيارك لمكان العمل بعد الاختصاص، حدد العوامل المؤثرة في قرارك:

العوامل	١	٢	٣	٤	٥
حفاظاً على العلاقات الاجتماعية					
الشعور بالانتماء للمجتمع					
نقص المتخصصين					
فرص أفضل لتعلم الأطفال					
فرصة للشريك (عمل/دراسة)					
فرصة كسب المال أكبر					
لإكمال مراحل التعليم					
استقرار وأمن أكثر					
تفضيلاً لنمط الحياة					
جودة الحياة العمليّة					
